# Screening of Litter-Size-Associated SNPs in *NOX4, PDE11A* and *GHR* Genes of Sheep

**DOI:** 10.3390/ani14050767

**Published:** 2024-02-29

**Authors:** Jiajun Li, Yiming Gong, Xiangyu Wang, Xiaoyun He, Xiaolong He, Mingxing Chu, Ran Di

**Affiliations:** 1 State Key Laboratory of Animal Biotech Breeding, Institute of Animal Science, Chinese Academy of Agricultural Sciences (CAAS), Beijing 100193, China; 82101225441@caas.cn (J.L.); 82101215374@caas.cn (Y.G.); wangxiangyu@caas.cn (X.W.); hexiaoyun@caas.cn (X.H.); 2 Inner Mongolia Academy of Agricultural and Animal Husbandry Sciences, Hohhot 010031, China; hexiaolong1983@163.com

**Keywords:** sheep, *NOX4*, *PDE11A*, litter size, molecular marker

## Abstract

**Simple Summary:**

*NOX4*, *PDE11A* and *GHR* have previously been screened as candidate genes for litter size in sheep. Therefore, it is necessary to validate these genes in the sheep population and identify loci that are associated with litter size. In this study, the results of association analysis showed that c.1057-4C > T in *NOX4* and c.1983C > T in *PDE11A* were significantly associated with litter size in Small Tail Han sheep, and their effects were independent of each other. In summary, this study provided two new genetic markers for improving litter size in sheep.

**Abstract:**

In previous studies, *NOX4*, *PDE11A* and *GHR* genes have been screened as important candidate genes for litter size in sheep by using the GWAS method; however, neither their effects on litter size nor the loci associated with litter size have been identified. In this study, three candidate loci (c.1057-4C > T in *NOX4*, c.1983C > T in *PDE11A* and c.1618C > T in *GHR*) were first screened based on our previous resequencing data of 10 sheep breeds. After the three loci were genotyped using Sequenom MassARRAY technology, we carried out population genetics analysis on the three loci and performed association analysis between the polymorphism of the three loci and the litter size of sheep. The results of population genetics analysis suggested that c.1057-4C > T in *NOX4* and c.1983C > T in *PDE11A* may be subject to natural or artificial selection. The results of association analysis indicated that litter size was significantly associated with c.1057-4C > T in *NOX4* and c.1983C > T in *PDE11A* (*p* < 0.05) in Small Tail Han sheep, and there was no significant interaction effect between the two loci on the litter size. In summary, c.1057-4C > T in *NOX4* and c.1983 C > T in *PDE11A* can be considered candidate molecular markers for improving litter size in sheep.

## 1. Introduction

Increasing litter size is one of the most effective ways to improve the economic benefits of sheep farms. However, as litter size is a trait with low heritability, it is difficult to improve in a short time period through conventional breeding methods. The marker selection breeding method is more effective for litter size improvement. Nevertheless, very few of the major genes and markers for litter size are currently known, so it is essential to explore new genetic markers for accelerating the genetic progress of litter size in sheep.

Through resequencing the genome of 248 sheep from 36 local breeds and 6 improved breeds around the world, Li et al. [1] screened reproductive candidate genes such as the reduced nicotinamide adenine dinucleotide phosphate oxidase 4 gene (*NOX4*) and the 11th member of the phosphodiesterase family gene (*PDE11A*) by simultaneously using genome-wide association study (GWAS) analysis and selection signal analysis. It was found that *NOX4* is highly expressed in the follicles of rats [2]. Activated *NOX4* can produce excess reactive oxygen species (ROS) and lead to sustained oxidative stress, impaired oocyte quality or follicular atresia in the ovary [3,4]. Furthermore, ROS was involved in the transduction of growth factors and played a key role in the ovulatory signal cascade of rodents [5], suggesting that ROS is an important regulatory factor during the ovulation process of animals. The *PDE11A* gene can catalyze the hydrolysis of cAMP [6]. In the animal ovary, cAMP acts as the second messenger for follicle-stimulating hormone (FSH) and luteinizing hormone (LH) receptors [7,8], and plays a central role in all stages of follicular development and ovulation [9]. Similarly, through GWAS analysis of five prolific and one low-fertility sheep breed, Xu et al. [10] screened the growth hormone receptor (*GHR*) as a candidate gene for litter size in sheep. *GHR* was also highly expressed in reproductive-related tissues such as the ovary and uterus. The GHR is located on the surface of early follicles, which can directly regulate the formation of late follicles by binding to the growth hormone (GH) [11]. Bachelot et al. found that the *GHR* gene is involved in the regulation of estrogen secretion, follicular growth and development, ovulation number and litter size in mice [12]. In addition, there were fewer follicles in *GHR*-knockout mice than in wild mice [11]. These results suggested that the above three candidate genes were closely related to follicular development and ovulation; however, the effect of these three genes on litter size in sheep remains unclear.

## 2. Materials and Methods

### 2.1. Animal Preparation and Sample Collection

All the sheep breeds that were analyzed in this experiment were divided into a polytocous group and a monotocous group. The polytocous group included Hu sheep (*n* = 96, Xuzhou City, Jiangsu Province, China), Cele Black sheep (*n* = 96, Qira County, Hotan Prefecture, Xinjiang Uygur Autonomous Region, China) and Small Tail Han sheep (*n* = 384, Heze City, Shandong Province, China). The monotocous group included Sunit sheep (*n* = 96, Urad Front Banner, Bayannaoer City, Inner Mongolia Autonomous Region, China) and Bamei mutton sheep (*n* = 96, Linhe District, Bayannaoer, Inner Mongolia Autonomous Region, China). The litter size information was recorded in detail in three parities of Small Tail Han sheep. However, it was difficult to obtain litter size records of all three parities in other sheep populations. Jugular vein blood was collected from all these ewes.

### 2.2. DNA Extraction

DNA was extracted from blood samples of the above sheep using a DNA extraction Kit (Beijing, China, Tiangen Biochemical Technology Co., Ltd., DP304-03). The concentration of DNA samples was determined by using a NanoDrop 2000 (Thermo Scientific, Madison, WI, USA), and the quality of DNA was detected through 1.5% agarose gel electrophoresis.

### 2.3. Selection of Candidate Loci in Three Genes

These three genes (*NOX4*, *PDE11A* and *GHR*) were selected as candidate genes to analyze their association with sheep litter size. First, based on our previous resequencing data of 10 sheep breeds [13], the polymorphic loci in these three genes were screened between the high-fecundity group (Hu sheep, Cele Black sheep, Small Tail Han sheep and Australian Merino sheep) and the low-fecundity group (Sunit sheep, Bamei mutton sheep, Tan sheep, Prairie Tibetan sheep, Valley Tibetan sheep, Oula sheep and Bayinbuluke sheep). Then, the allele frequency of each polymorphic locus in these 10 breeds and the population differentiation coefficient (F_st_) of each locus between the two groups were calculated. Next, the three candidate loci (c.1057-4C > T in *NOX4*, c.1983C > T in *PDE11A* and c.1618C > T in *GHR*) were screened based on their potential effects on the sequence and structure of protein and their F_st_ values (their F_st_ values need to meet any one of the following conditions: ① non-synonymous mutations or SNPs located in a splice region or regulatory region with F_st_ > 0.05; ② mutations in intron with F_st_ > 0.15).

### 2.4. Genotyping

First, according to the sheep’s *NOX4*, *PDE11A* and *GHR* gene sequences in GenBank ARS-UI_Ramb_v2.0 (accession nos.: NC_056069.1, NC_056074.1, NC_056055.1), single-base extension primers for three loci (c.1057-4C > T in *NOX4*, c.1983C > T in *PDE11A* and c.1618C > T in *GHR*) were designed using the MassARRAY Assay Design v.3.1. These three primers are shown in Table 1, which were synthesized by Beijing Compass Biotechnology Co., Ltd. (Beijing, China). Next, Sequenom MassARRAY^®^SNP technology was used to genotype the three loci in all five breeds (Small Tail Han sheep, Hu sheep, Cele Black sheep, Sunit sheep and Bamei mutton sheep). We referred to previous studies for the detailed process of genotyping [14,15].

### 2.5. Statistical Analysis

The allele and genotype frequency, polymorphism information content (*PIC*), heterozygosity (*He*) and number of effective alleles (*Ne*) were calculated using the following formulae:

(1) $$PIC=1-\sum_{i=1}^{n}{p_{i}}^{2}-\sum_{i=1}^{n-1}\;\sum_{j=i+1}^{n}2{p_{i}}^{2}{p_{j}}^{2}$$

(2) $$He=1-\sum_{i=1}^{n}{p_{i}}^{2}\;$$

(3) $$Ne=1/\;\sum_{i=1}^{n}{p_{i}}^{2}\;$$

where *n* is the number of alleles, *p_i_* is the allele frequency of the *i*th allele and *p_j_* is the allele frequency of the *j*th allele.

The chi-square test was used to detect whether the genotype distribution of each locus deviated from Hardy–Weinberg equilibrium. The association of litter size with the genotypes of three loci was analyzed using the linear model:

y=μ+P+G1+G2+G3+e

the least-squares means for multiple comparisons of litter size among the different genotypes in Small Tail Han sheep. *y* is the phenotypic value of litter size, *μ* is the population mean, *P* is the fixed parity effect, *G1* is the fixed effect for candidate SNP in *NOX4*, *G2* is the fixed effect for candidate SNP in *PDE11A*, *G3* is the fixed effect for candidate SNP in *GHR*, and *e* is the random error effect of each observation. Meanwhile, we also examined the interaction effect between each two loci, in order to check whether the interaction between candidate loci needs to be supplemented in the model. However, we did not find a significant interaction between the candidate loci. Analysis was performed using R software (avo, Version 4.0.3).

### 2.6. Protein Functional Domain and Interaction Networks Analysis

For SNP loci that were significantly associated with litter size, we further analyzed the location of the SNPs in the gene and their potential influence on nearby protein functional domains. In addition, in order to understand what kind of interaction network, including the candidate proteins, regulates follicular development, the interaction networks of the candidate proteins were predicted using the STRING database v.12.0 (https://cn.string-db.org/, accessed on 20 November 2023) [16].

## 3. Results

### 3.1. Genotyping and Population Genetic Analysis of Candidate SNPs in NOX4, PDE11A and GHR Genes

**Figure 1:**
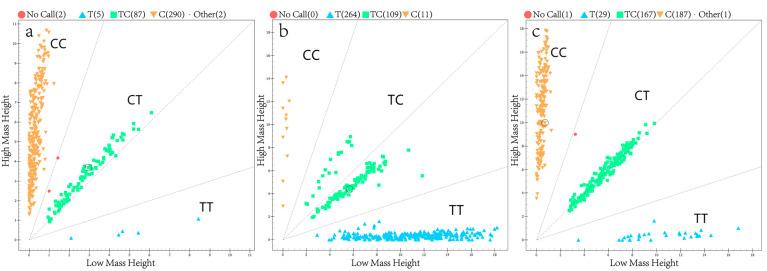
Genotyping results of candidate SNPs in *NOX4*, *PDE11A* and *GHR* genes using MassARRAY^®^SNP system. (**a**) c.1057-4C > T in *NOX4*; (**b**) c.1983C > T in *PDE11A*; (**c**) c.1618C > T in *GHR*.

The candidate SNPs (c.1057-4C > T in *NOX4*, c.1983C > T in *PDE11A* and c.1618C > T in *GHR*) were genotyped in all ewe samples, and the results are shown in Figure 1. It can be seen that there were three genotypes in each SNP locus, and three genotypes could be well distinguished by the MassARRAY system.

The results of population genetic analysis are summarized in Table 2. The c.1057-4C > T locus of the *NOX4* gene displayed a moderate polymorphism in Bamei mutton sheep (0.25 ≤ *PIC* < 0.5) and a low polymorphism in Small Tail Han sheep, Hu sheep, Cele Black sheep and Sunit sheep (*PIC* < 0.25). The c.1983 C > T locus of the *PDE11A* gene showed a moderate polymorphism (0.25 ≤ *PIC* < 0.5) in Hu sheep, Cele Black sheep and Bamei mutton sheep, and a low polymorphism (*PIC* < 0.25) in Small Tail Han sheep and Sunit sheep. The c.1618C > T locus of the *GHR* gene presented a moderate polymorphism (0.25 ≤ *PIC* < 0.5) in Small Tail Han sheep, Hu sheep, Sunit sheep and Bamei mutton sheep, and a low polymorphism (*PIC* < 0.25) in Cele Black sheep.

According to the results of the chi-square test, the c.1057-4C > T locus of the *NOX4* gene was in Hardy–Weinberg equilibrium (*p* > 0.05) in Small Tail Han sheep and Bamei mutton sheep only. The *PDE11A* gene c.1983 C > T locus was in Hardy–Weinberg equilibrium in Hu sheep, Cele Black sheep and Bamei mutton sheep (*p* > 0.05). The c.1618 C > T locus of the *GHR* gene was in Hardy–Weinberg equilibrium in all five sheep breeds (*p* > 0.05).

### 3.2. Association Analysis between Candidate Loci in NOX4, PDE11A, GHR and Litter Size of Small Tail Han Sheep

The association between the three SNP loci in *NOX4*, *PDE11A* and *GHR* genes with the litter size was analyzed in Small Tail Han sheep (Table 3). It is worth noting that there was a significant association between the polymorphism of the *NOX4* c.1057-4C > T locus and the litter size in the three parities of Small Tail Han sheep (*p* < 0.05). The litter size of ewes with the TT-genotype was significantly higher than that of ewes with the CT-genotype and CC-genotype (*p* < 0.05) in all three parities, and the litter size of CT-genotype ewes was also significantly higher than that of CC-genotype ewes (*p* < 0.05) in the first parity. For the *PDE11A* c.1983C > T locus, there was also a significant association between its polymorphism and the litter size of Small Tail Han sheep (*p* < 0.05). The litter size of TT-genotype ewes was significantly higher than those of CT- and CC-genotype ewes in all three parities (*p* < 0.05), and the litter size of CT-genotype ewes was also significantly higher than that of CC-genotype ewes in the second and third parities (*p* < 0.05). However, there was no significant association between *GHR* c.1618C > T locus and litter size in the Small Tail Han population (*p* > 0.05). Additionally, parity had a significant effect on the litter size of Small Tail Han sheep (*p* = 8.75 × 10^−12^ < 0.01).

### 3.3. Protein Structure and Interaction Network Analysis of NOX4 and PDE11A

**Figure 2:**
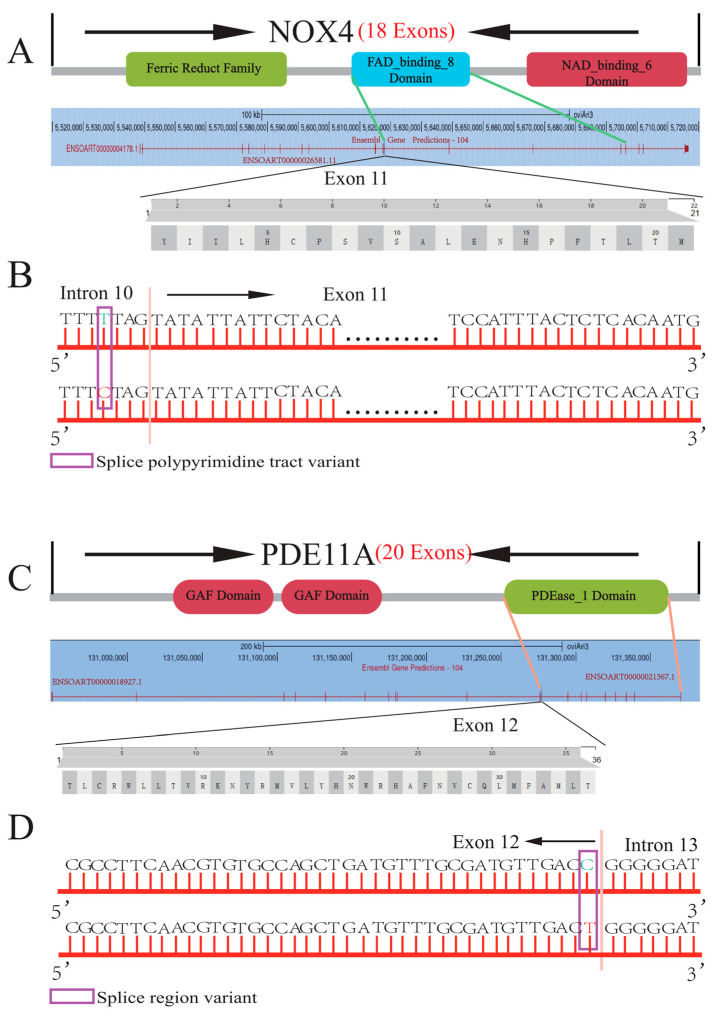
The location and functional domain of NOX4 c.1057-4C > T and PDE11A c.1983 C > T. (**A**) Functional domain of NOX4 and the location of exon 11. (**B**) Location of c.1057-4C > T in the NOX4 gene of sheep. (**C**) Functional domain of PDE11A and the location of exon 12. (**D**) Location of c.1983C > T in the PDE11A gene of sheep.

For the two SNPs (c.1057-4C > T and c.1983C > T) that were significantly associated with litter size, we further investigated the potential effect of the mutations on protein structure (Figure 2). Both SNPs belong to the splice polypyrimidine tract variant, which may lead to selective splicing. The SNP c.1057-4C > T is located at the posterior boundary of intron 10 in *NOX4* (Figure 2B), which might affect the splicing, composition and function of the domain including exon 11. Exon 11 of the *NOX4* gene is located in the FAD-binding_6 Domain (Figure 2A), which is the core catalytic part of ROS induction [17] and an important regulatory factor in the ovulation process of animals [18,19]. The SNP c.1983C > T is located at the last position of exon 12 in *PDE11A* (Figure 2D), which may affect the composition and function of the domain including exon 12. Exon 12 of the *PDE11A* gene is located in the PDEase_1 Domain, and this domain participates in catalyzing the hydrolysis of cAMP [20] and plays a central role in all stages of follicular development and ovulation [21].

**Figure 3:**
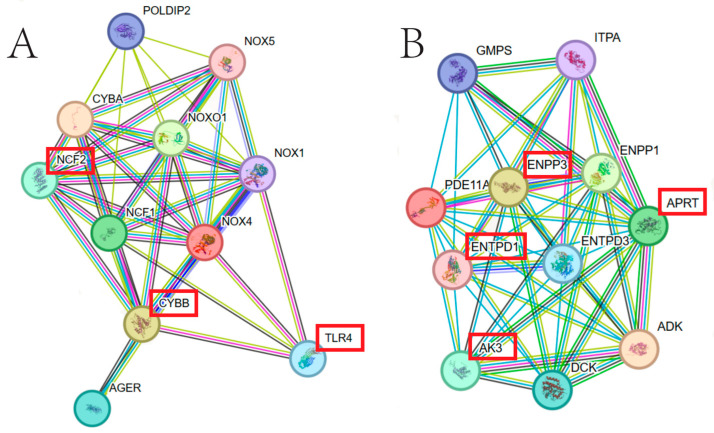
(**A**) Protein networks that closely interact with NOX4 in sheep, provided by the STRING database (version 12). (**B**) Protein networks that closely interact with PDE11A in sheep, provided by the STRING database (version 12). The proteins in the red box are closely related to ovulation and follicular development.

In order to further understand the biological functions of *NOX4* and *PDE11A* genes in the reproduction process of sheep, the protein networks that closely interact with them were constructed. Among the 10 strongest interacting proteins with NOX4 (Figure 3A,B), 3 (TLR4, NCF2 and CYBB) are crucial for follicular development as well as ovulation [22,23,24,25]. Among the 10 proteins that closely interact with PDE11A, 4 (AK3, APRT, ENPP3 and ENTPD1) are closely associated with oocyte development and ovulation [26,27,28].

## 4. Discussion

Through GWAS analysis and selection signal sweeps, Li et al. [1] and Xu et al. [10] screened key reproductive candidate genes, including *NOX4*, *PDE11A* and *GHR*. Therefore, these three genes are notable candidate genes, and their association with litter size should be studied further. In this study, based on our previous resequencing data of 10 sheep breeds, we screened three SNP loci in these three genes and further analyzed their association with litter size.

Population genetic analysis showed that c.1983 C > T in the *PDE11A* gene deviated from Hardy–Weinberg equilibrium in Small Tail Han sheep and Sunit sheep, and c.1057-4C > T in the *NOX4* gene deviated from Hardy–Weinberg equilibrium in Hu sheep, Cele Black sheep and Sunit sheep. The results suggest that both SNP loci might be under the influence of natural or artificial selection within these sheep breeds. *NOX4* gene expression regulates the production of ROS [29], whose excess generation can increase the incidence of diseases such as preeclampsia and lead to placental abruption or stillbirths [30]. Coincidentally, mutations in the *PDE11A* gene have also been found to be associated with preeclampsia, premature birth and stillbirths [31]. Thus, it is plausible that during the domestication and breeding processes of these sheep populations, these two SNP loci undergo strong artificial selection, aiming to minimize the occurrences of stillbirths and preterm births. Conversely, the SNP locus in the *GHR* gene maintained Hardy–Weinberg equilibrium across the five sheep breeds, suggesting that this particular locus may not have been influenced by either natural or artificial selection.

The association analysis demonstrated a significant relationship between the c.1057-4 C > T locus in the *NOX4* gene and litter size in Small Tail Han sheep. NOX4, a primary source of ROS production, is crucial for follicular maturation. Briefly, NOX4 plays an important role in the survival of granulosa cells and follicular development. However, the association between litter size and mutations in the gene has not been studied in sheep. The NOX4 protein consists of 594 amino acids that are encoded by 18 exons and contains three functional domains: Ferric reduct Family Domain (amino acids 73 to 220), FAD_binding_8 Domain (amino acids 321–432) and Ferric reductase NAD binding Domain (amino acids 438–577). Exon 11 is located in the FAD_binding_8 Domain, which is essential for electron transfer [32] and a central catalytic part of ROS production induction [17]. Unbalanced ROS levels may lead to oxidative stress and a significant decline in the number and quality of oocytes [18]. In addition, Gnainsky et al. found that reducing FAD levels can inhibit mitochondrial activity and oogenesis in oocytes [19]. It is worth noting that the c.1057-4 C > T mutation in *NOX4* is located at the anterior boundary adjacent to exon 11 and belongs to the splice polypyrimidine tract variant, which may affect the function of the FAD_binding_8 Domain through selective splicing and may change the concentration of ROS. Thus, follicular development, oocyte activity and oogenesis may ultimately be affected due to changes in ROS.

Similarly, according to the association analysis, c.1983C > T in *PDE11A* was significantly associated with litter size in Small Tail Han sheep. In mammalian oocytes, the meiotic cycle is influenced by the levels of cAMP and cGMP [33,34,35], which enhances the survival ability of granulosa cells [21]. The activity of cAMP-PDE is mainly provided by PDE11A and PDE8A [36]. In addition, mutations in *PDE11A* will affect the number of meiosis occurrences and oocyte ovulation [21]. However, we also did not find any study on the association between *PDE11A* gene mutations and litter size in animals. The PDE11A protein is encoded by 914 amino acids across 20 exons and consists of three functional domains: two GAF Domains (amino acids 195–348 and amino acids 380–536) and one PDEase_1 Domain (amino acids 643–878). Exon 12 is located in the PDEase_1 Domain, which is associated with the ability to catalyze cAMP hydrolysis [20]. The c.1983 C > T mutation in *PDE11A* is located at the last base of exon 12, which belongs to the splice region variant. Thus, it may affect the structure of the PDEase_1 Domain by alternative splicing and change the ability to catalyze the hydrolysis of cAMP. Ultimately, the above changes might affect oocyte meiosis, the survival ability of granulosa cells and the number of ovulations.

Based on the protein interaction network provided by the STRING database, we identified the 10 proteins that closely interact with NOX4. Among them, three proteins (TLR4, NCF2 and CYBB) were crucial for follicular development and ovulation. TLR4 is highly expressed in mouse cumulus cells and is involved in the proliferation of cumulus–oocyte complexes and ovulation. Moreover, its deficiency can affect ovulation and pregnancy rates [37]. Additionally, its level will increase in the induced PCOS mice [38]. The activity of NCF2 may lead to increased ROS accumulation, thereby reducing the development ability of oocytes [39,40]. Furthermore, Kuokkanen et al. found that elevated levels of *CYBB* and *NCF2* mRNA may potentially harm luteal cells [25]. Among the 10 proteins that are closely related to PDE11A, 4 (AK3, APRT, ENPP3, ENTPD1) play important roles in different periods from oocyte development to embryonic development. The expression of AK3 in oocytes indirectly regulates ATP levels and thus influences the activity of oocytes [41]. The activity of APRT varies across different periods of ovulation and embryonic development [26]. ENPP3 was identified as a new biological marker for tubal metaplasia [27] and is very important for morphological changes and inflammatory responses during ovulation and luteinization [42]. Kauffenstein et al. [43] found that the mice carrying a mutation in *ENTPD1* had a smaller litter size compared to the wild type. In summary, these networks further confirm the relationship between candidate genes and follicular development and litter size. However, the interplay among these genes and how they synergistically regulate follicular development and ovulation require further exploration.

## 5. Conclusions

The results of association analysis indicated that litter size was significantly associated with c.1057-4C > T in *NOX4* and c.1983C > T in *PDE11A* (*p* < 0.05) in Small Tail Han sheep, and there was no significant interaction effect between the two loci on the litter size. Therefore, these two loci can be considered candidate molecular markers for improving litter size in sheep.

## Figures and Tables

**Table 1:** The primer sequences for MassARRAY analysis.

Loci	Primer Sequence (5′-3′)
c.1057-4	F-ACGTTGGATGTTGAACTTCTTTCTTGGTC
	R-ACGTTGGATGTCTAATGCAGACACACTGGG
	EXT-GGGCAATGTAGAATAATATACTA
c.1983	F-ACGTTGGATGTGTGCCAGCTGATGTTTGCG
	R-ACGTTGGATGTAGGTCCTCACAAGTGACAG
	EXT-ACGTCACCAAACACACTTAC
c.1618	F-ACGTTGGATGCAGAAGTAAGCGCTGTCCAC
	R-ACGTTGGATGCCCCAGGCCAAAAGAATAAG
	EXT-TCACACCCAGCCAAGCA

**Table 2:** Population genetic analysis of candidate loci of *NOX4*, *PDE11A* and *GHR* genes in five sheep breeds.

Gene	SNPs	Breeds	*PIC*	*He*	*Ne*	χ^2^ (*p*)
*NOX4*	c.1057-4C > T	Small Tail Han sheep	0.20	0.23	1.3	0.30
		Hu sheep	0.11	0.11	1.13	0.00
		Cele Black sheep	0.18	0.20	1.24	0.00
		Sunit sheep	0.18	0.20	1.24	0.01
		Bamei mutton sheep	0.30	0.38	1.60	0.32
*PDE11A*	c.1983C > T	Small Tail Han sheep	0.07	0.08	1.08	0.02
		Hu sheep	0.37	0.49	1.97	0.83
		Cele Black sheep	0.25	0.30	1.42	0.10
		Sunit sheep	0.09	0.10	1.10	0.00
		Bamei mutton sheep	0.32	0.39	1.65	0.40
*GHR*	c.1618C > T	Small Tail Han sheep	0.33	0.42	1.72	0.44
		Hu sheep	0.36	0.47	1.87	0.51
		Cele Black sheep	0.21	0.24	1.32	0.52
		Sunit sheep	0.32	0.40	1.68	0.44
		Bamei mutton sheep	0.25	0.30	1.42	0.36

**Table 3:** Least-squares means and standard deviations of litter size for different genotypes in Small Tail Han sheep.

Gene	SNPs	Genotypes	First Parity	Second Parity	Third Parity
			Litter Size	Litter Size	Litter Size
*NOX4*	c.1057-4C > T	CC	2.13 ± 0.08 (261) ^c^	2.43 ± 0.07(193) ^bc^	2.55 ± 0.17 (56) ^bc^
		CT	2.40 ± 0.09 (79) ^b^	2.45 ± 0.11 (68) ^b^	2.62 ± 0.52 (21) ^b^
		TT	3.80 ± 0.63 (5) ^a^	3.50 ± 0.29 (5) ^a^	3.60 ± 0.67 (5) ^a^
*PDE11A*	c.1983C > T	CC	1.80 ± 0.43 (10) ^bc^	1.63 ± 0.52 (8) ^c^	1.67 ± 0.46 (6) ^c^
		CT	1.93 ± 0.20 (72) ^b^	2.33 ± 0.60 (45) ^b^	2.58 ± 0.50 (19) ^b^
		TT	2.30 ± 0.62 (265) ^a^	2.61 ± 0.76 (201) ^a^	3.07 ± 0.74 (70) ^a^
*GHR*	c.1618C > T	CC	2.25 ± 0.07 (168)	2.50 ± 0.08 (135)	2.98 ± 0.15 (54)
		CT	2.20 ± 0.06 (151)	2.38 ± 0.08 (120)	2.88 ± 0.16 (41)
		TT	2.04 ± 0.16 (27)	2.62 ± 0.33 (13)	3.00 ± 0.00 (2)

NOTE: The number in brackets denotes the number of ewes with the kind of genotype. Different lower-case letters next to the litter size mean that there are significant differences between genotypes (*p* < 0.05).

## Data Availability

The data presented in this study are available in the article.

## References

[1] Li X., Yang J., Shen M., Xie X.-L., Liu G.-J., Xu Y.-X., Lv F.-H., Yang H., Yang Y.-L., Liu C.-B. (2020). Whole-genome resequencing of wild and domestic sheep identifies genes associated with morphological and agronomic traits. Nat. Commun..

[2] Li Y., Xu J., Li L., Bai L., Wang Y., Zhang J., Wang H. (2022). Inhibition of nicotinamide adenine dinucleotide phosphate oxidase 4 attenuates cell apoptosis and oxidative stress in a rat model of polycystic ovary syndrome through the activation of Nrf-2/HO-1 signaling pathway. Mol. Cell. Endocrinol..

[3] Gong Y., Luo S., Fan P., Jin S., Zhu H., Deng T., Quan Y., Huang W. (2020). Growth hormone alleviates oxidative stress and improves oocyte quality in Chinese women with polycystic ovary syndrome: A randomized controlled trial. Sci. Rep..

[4] Zhang Y., Murugesan P., Huang K., Cai H. (2020). NADPH oxidases and oxidase crosstalk in cardiovascular diseases: Novel therapeutic targets. Nat. Rev. Cardiol..

[5] Shkolnik K., Tadmor A., Ben-Dor S., Nevo N., Galiani D., Dekel N. (2011). Reactive oxygen species are indispensable in ovulation. Proc. Natl. Acad. Sci. USA.

[6] Petersen T.S., Stahlhut M., Andersen C.Y. (2015). Phosphodiesterases in the rat ovary: Effect of cAMP in primordial follicles. Reproduction.

[7] Brucato S., Bocquet J., Villers C. (2002). Regulation of glypican-1, syndecan-1 and syndecan-4 mRNAs expression by follicle-stimulating hormone, cAMP increase and calcium influx during rat Sertoli cell development. Eur. J. Biochem..

[8] Pan B., Li J. (2019). The art of oocyte meiotic arrest regulation. Reprod. Biol. Endocrinol..

[9] Zhang P., Louhio H., Tuuri T., Sjöberg J., Hreinsson J., Telfer E.E., Hovatta O. (2004). In vitro effect of cyclic adenosine 3′, 5′-monophosphate (cAMP) on early human ovarian follicles. J. Assist. Reprod. Genet..

[10] Xu S.-S., Gao L., Xie X.-L., Ren Y.-L., Shen Z.-Q., Wang F., Shen M., Eyϸórsdóttir E., Hallsson J.H., Kiseleva T. (2018). Genome-Wide association analyses highlight the potential for different genetic mechanisms for litter size among sheep breeds. Front. Genet..

[11] Han L., Tian H., Guo X., Zhang L. (2022). Regulation of ovarian function by growth hormone: Potential intervention of ovarian aging. Front. Endocrinol..

[12] Bachelot A., Monget P., Imbert-Bolloré P., Coshigano K., Kopchick J.J., Kelly P.A., Binart N. (2002). Growth hormone is required for ovarian follicular growth. Endocrinology.

[13] Pan Z., Li S., Liu Q., Wang Z., Zhou Z., Di R., Miao B., Hu W., Wang X., Hu X. (2018). Whole-genome sequences of 89 Chinese sheep suggest role of RXFP2 in the development of unique horn phenotype as response to semi-feralization. Gigascience.

[14] Romar R., De Santis T., Papillier P., Perreau C., Thélie A., Dell’Aquila M.E., Mermillod P., Dalbiès-Tran R. (2011). Expression of maternal transcripts during bovine oocyte in vitro maturation is affected by donor age. Reprod. Domest. Anim..

[15] Zhou M., Pan Z., Cao X., Guo X., He X., Sun Q., Di R., Hu W., Wang X., Zhang X. (2018). Single nucleotide polymorphisms in the HIRA gene affect litter size in Small Tail Han sheep. Animals.

[16] von Mering C., Jensen L.J., Snel B., Hooper S.D., Krupp M., Foglierini M., Jouffre N., Huynen M.A., Bork P. (2005). STRING: Known and predicted protein-protein associations, integrated and transferred across organisms. Nucleic Acids Res..

[17] Ceccon M., Millana Fananas E., Massari M., Mattevi A., Magnani F. (2017). Engineering stability in NADPH oxidases: A common strategy for enzyme production. Mol. Membr. Biol..

[18] Wang L., Tang J., Wang L., Tan F., Song H., Zhou J., Li F. (2021). Oxidative stress in oocyte aging and female reproduction. J. Cell. Physiol..

[19] Gnainsky Y., Zfanya N., Elgart M., Omri E., Brandis A., Mehlman T., Itkin M., Malitsky S., Adamski J., Soen Y. (2021). Systemic regulation of host energy and oogenesis by microbiome-derived mitochondrial coenzymes. Cell Rep..

[20] Ramos Leal G., Santos Monteiro C.A., Souza-Fabjan J.M.G., de Paula Vasconcelos C.O., Garcia Nogueira L.A., Reis Ferreira A.M., Varella Serapião R. (2018). Role of cAMP modulator supplementations during oocyte in vitro maturation in domestic animals. Anim. Reprod. Sci..

[21] Shrestha K., Meidan R. (2018). The cAMP-EPAC pathway mediates PGE2-Induced FGF2 in bovine granulosa cells. Endocrinology.

[22] Xu J., Bao X., Peng Z., Wang L., Du L., Niu W., Sun Y. (2016). Comprehensive analysis of genome-wide DNA methylation across human polycystic ovary syndrome ovary granulosa cell. Oncotarget.

[23] Piersanti R.L., Horlock A.D., Block J., Santos J.E.P., Sheldon I.M., Bromfield J.J. (2019). Persistent effects on bovine granulosa cell transcriptome after resolution of uterine disease. Reproduction.

[24] Hirata T., Osuga Y., Hamasaki K., Hirota Y., Nose E., Morimoto C., Harada M., Takemura Y., Koga K., Yoshino O. (2007). Expression of toll-like receptors 2, 3, 4, and 9 genes in the human endometrium during the menstrual cycle. J. Reprod. Immunol..

[25] Kuokkanen S., Polotsky A.J., Chosich J., Bradford A.P., Jasinska A., Phang T., Santoro N., Appt S.E. (2016). Corpus luteum as a novel target of weight changes that contribute to impaired female reproductive physiology and function. Syst. Biol. Reprod. Med..

[26] Braude P.R., Monk M., Pickering S.J., Cant A., Johnson M.H. (1989). Measurement of HPRT activity in the human unfertilized oocyte and pre-embryo. Prenat. Diagn..

[27] Aliagas E., Vidal A., Torrejón-Escribano B., Taco Mdel R., Ponce J., de Aranda I.G., Sévigny J., Condom E., Martín-Satué M. (2013). Ecto-nucleotidases distribution in human cyclic and postmenopausic endometrium. Purinergic Signal..

[28] Brochiero E., Coady M.J., Klein H., Laprade R., Lapointe J.-Y. (2001). Activation of an ATP-dependent K^+^ conductance in Xenopus oocytes by expression of adenylate kinase cloned from renal proximal tubules. Biochim. Biophys. Acta (BBA)—Biomembr..

[29] Rozycki M., Bialik J.F., Speight P., Dan Q., Knudsen T.E.T., Szeto S.G., Yuen D.A., Szászi K., Pedersen S.F., Kapus A. (2016). Myocardin-related transcription factor regulates Nox4 protein expression. J. Biol. Chem..

[30] Shi X., Xiao D., Hu X.-Q., Huang X., Zhou J., Wilson S.M., Yang S., Zhang L. (2013). Chronic hypoxia during gestation enhances uterine arterial myogenic tone via heightened oxidative stress. PLoS ONE.

[31] Huan W., Dan L., Can-feng S., Liang L., Jing L., Hui C. (2022). Association of monogenic hypertension related pathogenic genes with preeclampsia. Chin. J. Hypertens..

[32] Bánfi B., Molnár G., Maturana A., Steger K., Hegedûs B., Demaurex N., Krause K.H. (2001). A Ca^2+^-activated NADPH oxidase in testis, spleen, and lymph nodes. J. Biol. Chem..

[33] Sela-Abramovich S., Galiani D., Nevo N., Dekel N. (2008). Inhibition of rat oocyte maturation and ovulation by nitric oxide: Mechanism of action. Biol. Reprod..

[34] Prasad S., Tiwari M., Tripathi A., Pandey A.N., Chaube S.K. (2015). Changes in signal molecules and maturation promoting factor levels associate with spontaneous resumption of meiosis in rat oocytes. Cell Biol. Int..

[35] Tiwari M., Chaube S.K. (2016). Moderate increase of reactive oxygen species triggers meiotic resumption in rat follicular oocytes. J. Obstet. Gynaecol. Res..

[36] Bergeron A., Guillemette C., Sirard M.A., Richard F.J. (2017). Active 3′-5′ cyclic nucleotide phosphodiesterases are present in detergent-resistant membranes of mural granulosa cells. Reprod. Fertil. Dev..

[37] Gonçalves J.P., Magalhães B.A., Campos-Junior P.H.A. (2022). Contrasting effects of the Toll-like receptor 4 in determining ovarian follicle endowment and fertility in female adult mice. Zygote.

[38] Wang D., Weng Y., Zhang Y., Wang R., Wang T., Zhou J., Shen S., Wang H., Wang Y. (2020). Exposure to hyperandrogen drives ovarian dysfunction and fibrosis by activating the NLRP3 inflammasome in mice. Sci. Total Environ..

[39] Tamura H., Takasaki A., Miwa I., Taniguchi K., Maekawa R., Asada H., Taketani T., Matsuoka A., Yamagata Y., Shimamura K. (2008). Oxidative stress impairs oocyte quality and melatonin protects oocytes from free radical damage and improves fertilization rate. J. Pineal Res..

[40] Lai Q., Xiang W., Li Q., Zhang H., Li Y., Zhu G., Xiong C., Jin L. (2018). Oxidative stress in granulosa cells contributes to poor oocyte quality and IVF-ET outcomes in women with polycystic ovary syndrome. Front. Med..

[41] Dzeja P.P., Terzic A. (1998). Phosphotransfer reactions in the regulation of ATP-sensitive K^+^ channels. FASEB J..

[42] Li F.X., Yu J.J., Liu Y., Miao X.P., Curry T.E. (2017). Induction of Ectonucleotide Pyrophosphatase/Phosphodiesterase 3 during the Periovulatory Period in the Rat Ovary. Reprod. Sci..

[43] Kauffenstein G., Pelletier J., Lavoie E.G., Kukulski F., Martín-Satué M., Dufresne S.S., Frenette J., Ribas Fürstenau C., Sereda M.J., Toutain B. (2014). Nucleoside triphosphate diphosphohydrolase-1 ectonucleotidase is required for normal vas deferens contraction and male fertility through maintaining P2X1 receptor function. J. Biol. Chem..

